# The Small RNA DsrA Influences the Acid Tolerance Response and Virulence of *Salmonella enterica* Serovar Typhimurium

**DOI:** 10.3389/fmicb.2016.00599

**Published:** 2016-04-26

**Authors:** Daniel Ryan, Urmesh K. Ojha, Sangeeta Jaiswal, Chandrashekhar Padhi, Mrutyunjay Suar

**Affiliations:** Infection Biology Laboratory, School of Biotechnology, KIIT UniversityBhubaneswar, India

**Keywords:** sRNA, ATR, virulence, inflammation, mice, adhesion, invasion, motility

## Abstract

The Gram-negative, enteropathogen *Salmonella enterica* serovar Typhimurium (*S*. Typhimurium) is exposed to various stress conditions during pathogenesis, of which acid stress serves as a major defense mechanism in the host. Such environments are encountered in the stomach and *Salmonella* containing vacuole of phagocytic and non-phagocytic cells. It is only recently that small RNAs (sRNAs) have come to the forefront as major regulators of stress response networks. Consequently, the sRNA DsrA which regulates acid resistance in *Escherichia coli*, has not been characterized in the acid tolerance response (ATR) of *Salmonella.* In this study, we show *dsrA* to be induced two and threefold under adaptation and challenge phases of the ATR, respectively. Additionally, an isogenic mutant lacking *dsrA* (ΔDsrA) displayed lower viability under the ATR along with reduced motility, feeble adhesion and defective invasion efficacy *in vitro*. Expression analysis revealed down regulation of several *Salmonella* pathogenicity island-1 (SPI-1) effectors in ΔDsrA compared to the wild-type, under SPI-1 inducing conditions. Additionally, our *in vivo* data revealed ΔDsrA to be unable to cause gut inflammation in C57BL/6 mice at 72 h post infection, although intracellular survival and systemic dissemination remained unaffected. A possible explanation may be the significantly reduced expression of flagellin structural genes *fliC* and *fljB* in ΔDsrA, which have been implicated as major proinflammatory determinants. This study serves to highlight the role of sRNAs such as DsrA in both acid tolerance and virulence of *S*. Typhimurium. Additionally the robust phenotype of non-invasiveness could be exploited in developing SPI-I attenuated *S.* Typhimurium strains without disrupting SPI-I genes.

## Introduction

*Salmonella enterica* are a group of Gram-negative bacteria capable of causing clinical symptoms ranging from diarrhea to severe enteritis as well as life threatening systemic infections (Galán, 2001). During pathogenesis, *Salmonella* are exposed to an array of hostile conditions such as acidic pH, reactive oxygen species, bile salts, etc (Suar and Ryan, 2015). One such stress most frequently encountered is low pH and the ability of *Salmonella* to sense and respond to this is essential to its survival strategy. Salmonellae are exposed to acidic pH primarily in the stomach and within the *Salmonella* containing vacuole of phagocytic and non-phagocytic cells (Allam et al., 2012). This acidification serves as a major defense of the host and promotes the killing effects of other bactericidal mechanisms namely increased formation of phagolysosomes, activation of acid hydrolases and formation of free radical species. Following acid exposure, Salmonellae adopt different survival strategies collectively termed the acid tolerance response (ATR; Foster and Hall, 1990). Under this response, when *S. enterica* subspecies enterica Serovar Typhimurium (*S*. Typhimurium) cells are grown at sub-lethal pH (4.5–5.5) for one generation, they gain the ability to survive at normally lethal pH levels (Baik et al., 1996). During this adaptation, the cell synthesizes a number of acid shock proteins (ASPs) at moderate pH levels that provide protection on subsequent exposure to severe acid (Foster, 1993).

To date, a number of studies have characterized the roles of various protein coding genes and regulators associated with the ATR (Foster, 1993; Audia et al., 2001). However, there has been very little emphasis on the small RNA (sRNA) complement associated with such a response. It is of late that sRNAs have come to the forefront as master regulators within a cell and have been shown to be highly involved in stress response and virulence (Gong et al., 2011; Hébrard et al., 2012). They are generally 50–500 nucleotides in length, ubiquitous in all kingdoms of life and normally do not code for protein products (Ryan et al., 2015). They function by base pairing to target mRNAs and bring about either repression or activation by affecting mRNA stability or exposing the ribosome binding site, respectively. Binding may sometimes involve the RNA chaperone Hfq to facilitate the sRNA-mRNA interaction. Several sRNAs may also bind directly to proteins and modify their functions (Altuvia, 2007).

The 87 nucleotide sRNA DsrA present in both *Escherichia coli* (*E*. *coli*) and *Salmonella* has been shown to be a regulator of acid resistance in the former only (Lease et al., 2004). Additionally, DsrA has also been shown to be repressed in non-proliferating *S.* Typhimurium within fibroblasts suggesting a possible role in the extracellular environment (Ortega et al., 2012). Two targets regulated by DsrA in *E. coli* include the stress sigma factor *rpoS* and the histone-like nucleoid protein *hns*, both of which regulate genes involved in stress response and virulence (Nadim et al., 1998). Furthermore, *E. coli dsrA* mutants were found to be acid sensitive (Lease et al., 2004). To this effect, the present work was undertaken to characterize the role of DsrA in the ATR and virulence of the enteropathogen, *S*. Typhimurium.

## Materials and Methods

### Bacterial Strains and Growth Conditions

The strains and plasmids used in this study are listed in **Table 1**. Streptomycin (Sm; 50 μg/ml), kanamycin (Km; 50 μg/ml), chloramphenicol (Cm; 20 μg/ml) and ampicillin (Amp; 100 μg/ml) were added as appropriate to selected strains. Growth kinetics and ATR assays were performed in minimal *E* glucose medium (MEM; Vogel and Bonner, 1956). The medium was supplemented with dextrose (sterilized with 0.22 μm filter) at a concentration of 0.4% and casein hydrolysate (sterilized with 0.22 μm filter) at a concentration of 0.1% to allow for *S.* Typhimurium SB300 growth.

**Table 1:** Strains and plasmids used in this study.

	Description	Reference
**Strain**		
WT	*Salmonella enterica* serovar Typhimurium SB300	Pati et al., 2013
ΔDsrA	ΔDsrA::Km *S.* Typhimurium SB300 (Knockout strain)	This study
ΔInvC	(invC::aphT; *S.* Typhimurium SB300 (Knockout strain)	Pati et al., 2013
cDsrA	ΔDsrA::pZE12-*dsrA S.* Typhimurium SB300 (Complemented strain)	This study
**Plasmid**		
pKD46	aaaa red recombinase expressing plasmid (Amp^r^)	Datsenko and Wanner, 2000
pKD3	Plasmid containing FRT-flanked chloramphenicol resistance gene	Datsenko and Wanner, 2000
pKD4	Plasmid containing FRT-flanked kanamycin resistance gene	Datsenko and Wanner, 2000
pZE12-*luc*	sRNA complementing vector (Amp^r^)	Urban and Vogel, 2007
pZE12-*dsrA*	DsrA expressing vector (Amp^r^)	This study

Growth kinetics in MEM were determined by growing strains in triplicate over a period of 8 h at 37°C and 180 rpm. For the initial 2 h post subculture, OD_600_ measurements and plating of appropriate dilutions was done every 30 min. Subsequently, OD_600_ measurements and plating were performed every hour up to a total of 8 h.

Log-phase ATR was induced as described previously. (Baik et al., 1996). Briefly, a single colony of overnight grown *S.* Typhimurium SB300 culture in 5 ml MEM (37°C and 180 rpm) was sub-cultured (1:100 dilution) into 100 ml flasks and incubated until an OD_600_ of 0.4 was attained. Following this, cultures to be adapted were adjusted to pH 4.4 (±0.1) with 3 N HCl and incubated for 1 h. Adapted cultures were subsequently challenged at pH 3.1 (±0.1) as above and incubated for 1, 2, and 4 h post challenge. Colony forming units (CFUs) were calculated after plating appropriate dilutions on agar plates. Percent viability was calculated by dividing the CFU at different time points post challenge by CFU prior to challenge and multiplying the result by 100. ATR induction experiments were performed in triplicate.

### Construction of *dsrA* Mutant in *Salmonella* Typhimurium and Generation of Complemented Strain

All primers used in this study are listed in Supplementary Table S1. The *dsrA* deletion mutant was constructed using the lambda red recombinase system (Datsenko and Wanner, 2000). Knockout mutants were selected for Km resistance and confirmed by colony PCR using specifically designed confirmatory primers.

The complementation construct of *dsrA* was based on the pZE12-*luc* vector (Urban and Vogel, 2007). The *dsrA* gene was amplified from the genome of *S.* Typhimurium SB300 (Accession No. FQ312003.1) using primers dsraclF and dsraclR with n*pfu* special polymerase (Enzynomics, South Korea). The sense primer anneals to the +1 site of the sRNA gene leaving a 5′-blunt end in the PCR amplicon. The anti-sense primer adds an XbaI site to its 3′end. Following gel purification, the amplified product was digested with XbaI (New England Biolabs, USA) and subsequently gel extracted. The pZE12-*luc* vector was amplified with primers pLlacOB and pLlacOC to generate a linear fragment as mentioned above. plLacOC has a 5′ mono-phosphate required for cloning. Digestion with XbaI generates a fragment of ∼2.2 Kb that carries the PLlacO promoter (from the position –1), an ampicillin resistance cassette, a ColE1 replicon and a strong rrnB terminator, followed by the XbaI sticky end. XbaI digested products were ligated for 1 h at 22°C to yield the ligated construct pZE12-*dsrA*. The orientation of the insert was confirmed using the primer set dsraclF and pLlacOC. The construct was subsequently transformed into the isogenic *dsrA* mutant (ΔDsrA) and selected on Amp plates to yield the complemented strain (cDsrA).

### Quantitative Reverse Transcription PCR Assays

*Salmonella* Typhimurium RNA was extracted at pH 7.5 (prior to adaptation), pH 4.4 (1 h post adaptation) and pH 3.1 (1 h post challenge) from ATR cultures. Following RNase free DNase1 (Thermo Fisher Scientific, USA) treatment, cDNA was synthesized using Hi-cDNA Synthesis Kit (HIMEDIA, India). Quantitative reverse transcription polymerase chain reaction (qRT-PCR) was performed in triplicate using Kapa Sybr Fast qPCR Master Mix (2x; Kapa Biosystems, USA) with appropriately diluted cDNA templates. The guanylate monophosphate kinase gene (*gmk*) was used as an internal control after ensuring its suitability under acid stress.

For *Salmonella* pathogenicity island-1 (SPI-1) and flagellin gene expression, ΔDsrA and *S.* Typhimurium SB300 (WT) were grown in Luria-Bertani (LB) broth (0.3 M NaCl) and sub-cultured for 4 h (37°C, 120 rpm). Subsequent RNA extraction, purification, cDNA synthesis and qRT-PCR were performed as mentioned above. All assays were performed with three biological replicates in triplicate.

### Motility Assays

Motility assays were performed in triplicate on soft agar plates (0.3% w/v agar) prepared in LB. Briefly, 1 μl of bacterial culture was spotted at the center of each plate, incubated for 8 h at 37°C and the diameter of motile cell growth was measured.

### Adhesion and Invasion Assays

Adhesion and invasion assays were performed in HCT116 and HeLa colonic cell lines as described previously (Pati et al., 2013). In brief, colon epithelial cells were grown in Dulbecco’s modified Eagle’s medium (DMEM; Invitrogen, USA) supplemented with 10% fetal bovine serum (FBS) in 24-well plates at 37°C in a CO_2_ incubator until 80% confluent. Cells were washed with phosphate buffered saline (PBS) twice, followed by the addition of 500 μl supplemented DMEM (without antibiotic) prior to infection. Bacterial cultures were grown overnight in LB broth (0.3 M NaCl) at 37°C and 120 rpm with sub-culturing (1:20 dilution) into fresh medium until an OD_600_ of 0.6. Adhesion studies were performed by placing both 24-well plates and inoculum on ice for 15 min before infection. Infection was carried out at a multiplicity of infection (MOI) of 10 with subsequent incubation for half an hour on ice. Invasion assays were performed with a MOI of 10, followed by a 50 min incubation at 37°C and 5% CO_2_. Cells were subsequently washed twice with PBS and 500 μl gentamicin (100 μg/ml) containing DMEM was added and incubated for 2 h. Following incubation, cells were twice washed in PBS, lysed with 500 μl lysis solution (PBS containing 0.1% sodium desoxycholate) and suitable dilution plated on LB agar plates with appropriate antibiotics. Adhesion and invasion were calculated as percentages of the number of bacteria recovered, from the total bacteria inoculated. All assays were performed with three biological replicates in triplicate.

### Mice Infection and Histopathological Evaluation

This study utilized C57BL/6 mice that were maintained at the animal house of School of Biotechnology, KIIT University, Odisha, India. Six–eight weeks old Sm pre-treated mice were used in this study, as previously described (Pati et al., 2013). WT, ΔDsrA, and cDsrA were grown overnight in LB broth and subsequently sub-cultured (1:20) for 4 h. Mice were infected in groups of 5 (oral gavage) with ∼10^7^ CFU. Bacterial loads in the cecum content, mesenteric lymph node (MLN), spleen and liver were determined 72 h p.i. by plating suitable dilutions of homogenates on MacConkey agar plates supplemented with the appropriate antibiotics. Histopathological evaluation was performed as previously described (Pati et al., 2013). Briefly, Stained cryosections of the cecum were scored in a blinded manner by two pathologists on the basis of changes indicating sub-mucosal oedema, polymorphonuclear neutrophil (PMN) infiltration, goblet cell loss and epithelial ulceration (Barthel et al., 2003). Pathological scores ranged from 0 to 13 arbitrary units corresponding to levels of inflammation including intact intestines showing no inflammation (pathoscore 0); very mild inflammation (pathoscore 1–2); slight inflammation (pathoscore 3–4); intermediate inflammation (5–8); and significant inflammation (9–13).

### Statistical Analysis

All data represents triplicate experiments with mean ± standard deviation. Two-way Analysis of Variance (ANOVA) and *t*-tests were used to analyze significant differences. GraphPad Prism version 6.0 was used for all analyses.

### Ethical Statement

All *in vivo* experiments were performed according to the guidelines of the Institutional Animal Ethics Committee (IAEC), KIIT University bearing the approval number: KSBT/IAEC/2015/MEET-1/A2.

## Results

### *S*. Typhimurium ATR

**FIGURE 1:**
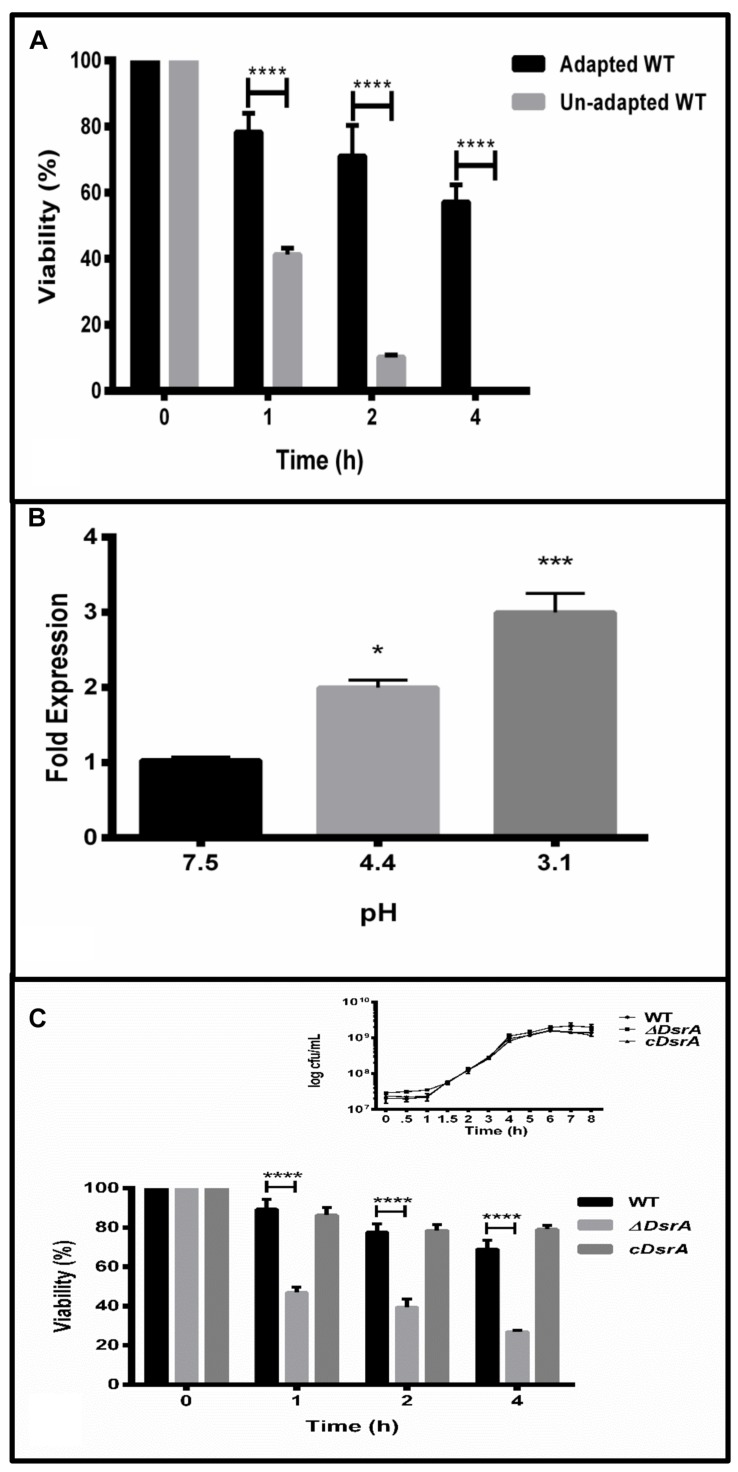
**(A)** The log-phase acid tolerance response (ATR) of *Salmonella* Typhimurium. Overnight grown cultures were sub-cultured into minimal EG medium (MEM) at a pH of 7.5 till OD_600_ of 0.4. Cultures to be adapted were adjusted to pH 4.4 ± 0.1 (3 N HCl) for 1 h. Un-adapted cultures were left untouched. All flasks were subsequently challenged by adjusting pH to 3.1 ± 0.1 (3 N HCl) and grown for 1, 2, and 4 h post challenge. Viable cells were determined by plating of appropriate dilutions on selective plates. **(B)** Quantitative reverse transcription polymerase chain reaction (qRT-PCR) of *dsrA* under normal (pH 7.5), adaptation (pH 4.4) and challenge (pH 3.1, 1 h), respectively. *gmk* (guanylate monophosphate kinase) was used as the internal control after verifying its suitability under the ATR. **(C)** ATR of WT, ΔDsrA and complemented strain (cDsrA). All experiments were performed in triplicate with data represented as mean ± SD. Statistical significance: ^∗^*P* < 0.05, ^∗∗∗^*P* < 0.001, ^∗∗∗∗^*P* < 0.0001.

Prior to determining the expression profile of *dsrA* under the ATR, WT was tested for its ability to mount an effective ATR in MEM. Both adapted (mid log phase cells grown for 60 min at pH4.4) and un-adapted (mid log phase) cells were tested for their ability to survive severe pH exposure (pH 3.1 ± 0.1) at various time points post acid challenge. As expected, adapted cells with their enhanced ability to survive harsh pH, displayed greater survival at all time points post challenge in contrast to normal un-adapted cells (**Figure 1A**). This result is in line with previously reported data in the literature (Bourret et al., 2008).

### *S*. Typhimurium *dsrA* Expression is Up-regulated under the ATR

The 87 bp sRNA DsrA has been reported to be a regulator of acid resistance in *E. coli* (Lease et al., 2004) and shares about 83% similarity at the nucleotide level with that of *S.* Typhimurium (Supplementary Figure S1). To determine the expression of *dsrA* under the ATR, qRT-PCR analysis revealed *dsrA* to be up-regulated two and threefold under acid adaptation and challenge, respectively, relative to levels at pH7.5 (**Figure 1B**). Guanylate monophosphate kinase (*gmk*) was used as the reference gene after ensuring its levels did not fluctuate under the observed conditions.

### Role of DsrA in the ATR of *S.* Typhimurium

To study the influence of DsrA on the ability of *S.* Typhimurium to induce an effective ATR, we compared the survival displayed by adapted cultures of ΔDsrA, WT and cDsrA to acid challenge as mentioned above after ensuring the growth of both ΔDsrA and cDsrA was not hampered (**Figure 1C**, inset). Interestingly, ΔDsrA was able to mount an ATR, however, there was a twofold decrease in viable cells at all time points post challenge, relative to both WT and cDsrA (**Figure 1C**).

### Motility Assays and Expression Analysis of Flagellin Structural Genes *fliC* and *fljB*

**FIGURE 2:**
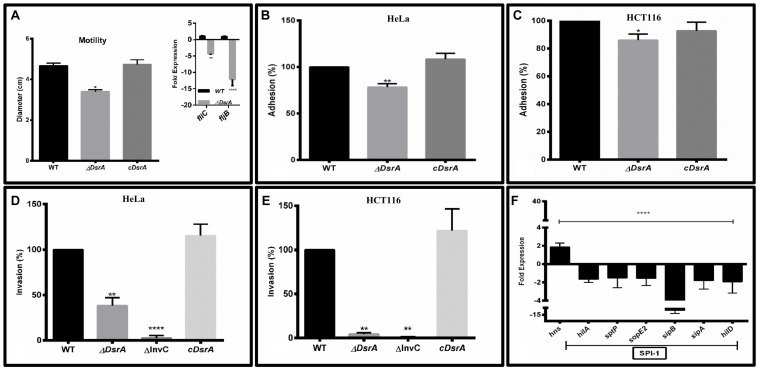
*****In vitro*** characterization of ΔDsrA. (A)** Motility assay of isogenic *dsrA* mutant ΔDsrA, cDsrA, and WT *S*. Typhimurium (SB300). qRT-PCR analysis of the flagellin structural genes *fliC* and *fljB* are shown in the inset. Guanylate monophosphate kinase (*gmk*) was used as an internal control. **(B–E)** Adhesion and invasion assays of ΔDsrA, invasion deficient negative control ΔInvC, cDsrA, and WT in HeLa and HCT116 cell lines. **(F)** qRT-PCR analysis of *hns* and *Salmonella* pathogenicity island 1 (SPI-1) associated genes in WT and ΔDsrA. *gmk* served as an internal control. All experiments were performed in triplicate with data represented as mean ± SD.Statistical significance: ^∗^*P* < 0.05, ^∗∗^*P* < 0.01, ^∗∗∗∗^*P* < 0.0001.

Motility assays of ΔDsrA, WT control and cDsrA indicated reduced motility in the mutant while the cDsrA remained comparable to the WT (**Figure 2A**). To further investigate this reduction in motility, we determined the relative expression of flagellin structural components *fliC* and *fljB*. qRT-PCR analysis revealed *fliC* and *fljB* to be down regulated 4 and 12-fold, respectively, in ΔDsrA relative to the WT strain, under SPI-1 inducing conditions (**Figure 2A** inset). The reduced expression of these genes accounts for the observed motility phenotype of ΔDsrA.

### ΔDsrA Displays Mildly Attenuated Adhesion, while Invasion is Highly Deficient *In Vitro*

To study the role of DsrA in adhesion to epithelial cells, both HeLa (cervical cancer line) and HCT116 (colon cancer line) cells were inoculated with ΔDsrA, cDsrA, and WT control. The *dsrA* mutant displayed mild attenuation in adhesion of about 86 and 78% in HCT116 and HeLa, respectively, relative to both WT and cDsrA which displayed comparable adhesion percentages (**Figures 2B,C**). We hypothesize this mild reduction may be due to an insufficiency in a number of factors including various fimbrae (Type 1, Curli, Pef, and Std) and pathogenicity island associated genes (*sipC*, *sipD*, *siiE*, and sipB, etc.) (Fàbrega and Vila, 2013).

To determine the role of DsrA in the context of bacterial invasion, both HCT116 and HeLa cells were inoculated with ΔDsrA and the invasion efficiency was compared to ΔInvC (invasion deficient negative control), cDsrA and WT control. ΔDsrA exhibited a significant lower invasion phenotype of approximately 4.5 and 38% in HCT116 and HeLa, respectively, relative to both WT and cDsrA (**Figures 2D,E**). The reduced invasion of the mutant strain may be attributed to a deficiency in the functioning of type 3 secretion system-1 (T3SS-1) which is necessary for bacterial invasion (Pati et al., 2013).

### Deletion of *dsrA* Altered the Expression Profile of Several Genes Associated with Invasion

To determine the role of DsrA in regulating SPI-1, we analyzed the expression of DsrA target *hns* along with a panel of SPI-1 master regulators and effectors in the ΔDsrA strain relative to the WT, under SPI-1 inducing conditions, using qRT-PCR (**Figure 2F**). This is based on the observation that H-NS has been shown to repress a number of pathogenicity islands in *Salmonella* (Fàbrega and Vila, 2013). The expression of *hns* was found to be up-regulated 1.8-fold in the mutant while the SPI-1 master regulator *hilA* was down-regulated 1.6-fold. Additionally, several effectors namely *sptP*, *sopE2*, *sipA*, and *SipB* were repressed along with another regulator *hilD* in ΔDsrA, thus further enforcing the reduced invasive phenotype.

### ΔDsrA Displayed Competitive Colonization But Was Unable to Cause Colitis in a Sm Pre-treated Mouse Model

**FIGURE 3:**
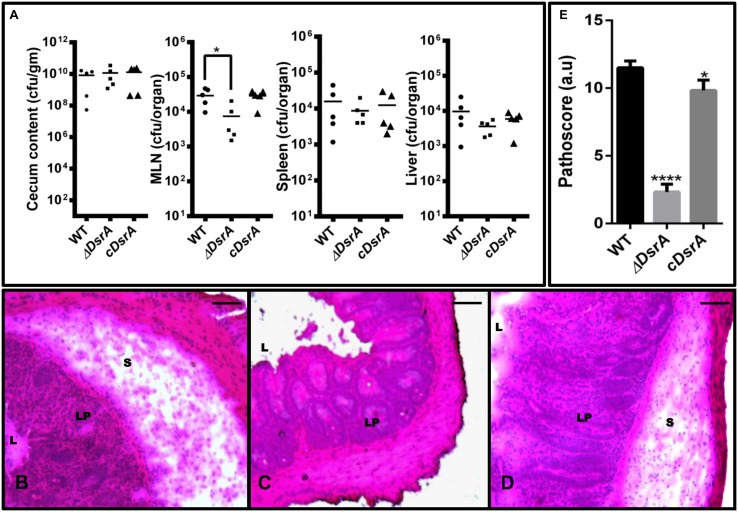
**Colonization and cecal inflammation by WT, ΔDsrA, and cDsrA in C57BL/6 mice. (A)** Streptomycin-pretreated mice were infected with the above strains, sacrificed 72 h p.i. and bacterial loads in the cecum, messenteric lymph node (MLN), liver and spleen were determined. **(B–D)** Histopathological assessment of the cecal tissue from animals infected with WT, ΔDsrA and cDsrA, respectively. **(E)** Analysis of cecal pathology by a semi-quantitative method as mentioned in the materials and methods. Bars, 200 μm. S, submucosa; L, lumen; Lp, lamina propria. Statistical significance: ^∗^*P* < 0.05, ^∗∗∗∗∗^*P* < 0.0001.

We compared the virulence profile of ΔDsrA to that of cDsrA and WT in C57BL/6 mice. In all cases, similar levels of colonization were observed with ΔDsrA displaying a mild decrease in MLN colonization (**Figure 3A**). Of note, ΔDsrA was unable to cause colitis displaying no inflammation, edema or loss of goblet cells 72 hpi. This is in stark contrast to that observed in the case of cDsrA and WT, both of which displayed severe inflammation, oedema, and loss of goblet cells at the same time interval (**Figures 3B–D**). Pathological scoring revealed cecum infected with ΔDsrA to have a pathoscore of 2.3 while that infected with WT and cDsrA was 11.5 and 9.8, respectively (**Figure 3**).

## Discussion

The ability of *Salmonella* to sense and adapt to changes in its environment is essential to its survival both in nature and within its host. One such stress that *Salmonella* must overcome is that of acid. The system that enables this is termed the ATR and it allows *Salmonella* to survive in normally lethal acidic pH levels following adaptation to mild acidic conditions (Foster, 1993; Garcia-del Portillo et al., 1993; Lin et al., 1995). This adaptation process involves the production of numerous ASPs and the up-regulation of systems that help in the maintenance of intracellular pH and consumption of excess protons within the bacterium (Audia et al., 2001; Ryan et al., 2015). Additionally, successful pathogenesis requires a bacterium to regulate certain host cell functions so as to be able to establish an infection. In *Salmonella*, genes belonging to SPIs play an important role in invasion of epithelial cells and intracellular survival. In addition to the above mentioned genes, sRNAs have recently been implicated in a number of stress and virulence pathways. Transcriptomics and genomics based approaches have predicted the existence of a number of sRNAs under various conditions, however, the vast majority remain functionally uncharacterized and thus, a complete strata of regulation seems to be lacking (Erdmann et al., 2001). One such sRNA, DsrA has been shown to regulate resistance to acid stress in *E. coli* and be repressed within intracellular Salmonellae within fibroblasts. (Lease and Belfort, 2000; Lease et al., 2004; Ortega et al., 2012). To this effect, we speculated a role for DsrA in the ATR of *S.* Typhimurium and characterized its effect on virulence.

The *dsrA* gene is induced under the *S.* Typhimurium ATR in MEM with maximum induction at pH 3.1. Further, deletion of *dsrA* was found to reduce the effectiveness of the ATR with lower percent viability at all three time points post acid challenge compared to wild-type SB300. This observation suggests a role for DsrA in the ATR of *Salmonella*, possibly through regulation of the stress response sigma factor *rpoS* (Lease et al., 2004), a first for any sRNA, although further work needs to be performed to confirm this hypothesis The link between acid survival and pathogenesis is an important characteristic for enteric pathogens such as *Salmonella*, which are constantly exposed to low pH during their course of infection in a host as well as in their habitat (Suar and Ryan, 2015). To this effect, we have further characterized the role of DsrA in the virulence of this pathogen.

ΔDsrA was found to exhibit slightly reduced motility and displayed a mild reduction in its ability to adhere to both HCT and HeLa intestinal epithelial cell lines. Intriguingly, the mutant displayed a significantly reduced ability to invade the aforementioned cell lines. The nucleoid protein and global silencer of transcription *hns*, has been shown to be repressed at the translational level by DsrA in *E. coli* although little is known as to whether this occurs by the enhancement of mRNA turnover or by the occlusion of ribosomes (Lease et al., 1998). H-NS in turn is a silencer of the SPI-1 master regulator *hilA* (Fàbrega and Vila, 2013) which is responsible for the induction of various effectors which are required for successful invasion of epithelial cells (Hapfelmeier et al., 2004). Thus, we suggest the sRNA DsrA mediates its effects on SPI-1 through this regulatory circuit which accounts for the highly reduced invasive phenotype. To validate this, we determined the expression levels of *hns* and several SPI-1 associated genes in ΔDsrA relative to wild-type under SPI-1 inducing conditions. Interestingly, we found *hns* levels to increase in a subtle but reproducible manner in accordance with what has already been reported (Lease et al., 1998), while *hilA* and several effector levels were repressed. The decrease in expression has a direct consequence of inhibiting invasion of epithelial cells.

Our *in vivo* data revealed ΔDsrA colonization of the cecum and other systemic organs to be comparable to that of wild-type SB300 at 72 hpi, however, there was considerable difference in the observed cecal pathology. ΔDsrA was found to be attenuated in developing gut inflammation at 72 hpi. This attenuation cannot be explained by a mere reduction in the expression of SPI-1 genes as earlier studies have shown *Salmonella* SPI-1 mutants to induce comparable cecal inflammation to the WT at the same time point (Hapfelmeier et al., 2005). On the other hand, several studies on *Salmonella* as a model for pathogenesis have revealed flagellin to play a major role in triggering proinflammatory responses in epithelial cells (Lyons et al., 2004). Double mutants of the flagellin structural genes *fliC* and *fljB* were found to be unable to induce a proinflammatory response (Zeng et al., 2003). Consequently, our expression analysis revealed significantly lower levels of expression of these two genes in ΔDsrA, suggesting a possible cause for the observed phenotype in addition to the reduced motility observed in the *dsrA* mutant.

To conclude, the enteric pathogen *Salmonella* must efficiently sense and respond to various environmental challenges, acidic pH being of prime importance. To achieve this, the bacterium is able to detect low pH through various two component systems namely PhoP/Q, EnvZ/OmpR, etc., followed by the initiation of a specific response termed the ATR (Bearson et al., 1998; Bang et al., 2000; Ryan et al., 2015). Furthermore, survival and adaptation to acidic pH is essential to the virulence of this pathogen. Our study has highlighted the role of the sRNA DsrA in the ATR of *Salmonella*. Additionally, we have shown DsrA to be required for the invasion of epithelial cell lines possibly through the *hns*, *hilA*, SPI-1 regulatory route. Virulence studies in mice demonstrated DsrA may be required for eliciting an inflammatory response mediated through possible regulatory effects on the flagellin structural genes *fliC* and *fljB*. However, it remains necessary to clarify the effect of DsrA on these genes. Successful *Salmonella* pathogenesis requires that the bacterium survive a plethora of stressors in its environment, a major one being acidic pH of the stomach and SCV. Additionally, there must be a coordinated regulation of various pathogenicity island associated genes to ensure successful invasion and survival within host cells (Fàbrega and Vila, 2013). Consequently, this work has served to highlight the importance of the link between acid survival and virulence with another strata of regulation namely, sRNAs being brought into the fold. Finally, the phenotype of non-invasiveness of ΔDsrA could be exploited for the development of SPI-1 attenuated strains without disrupting SPI-1 genes.

## Author Contributions

DR and MS conceived and designed the experiments. DR, UO, SJ, and CP performed the experiments. DR and MS analyzed and wrote the paper.

## Conflict of Interest Statement

The authors declare that the research was conducted in the absence of any commercial or financial relationships that could be construed as a potential conflict of interest.
