# Supplementary figures and images for: The Small RNA DsrA Influences the Acid Tolerance Response and Virulence of Salmonella enterica Serovar Typhimurium
Source: Front Microbiol. 2016 Apr 26;7:599. doi: 10.3389/fmicb.2016.00599 (PMC4844625; doi:10.3389/fmicb.2016.00599)

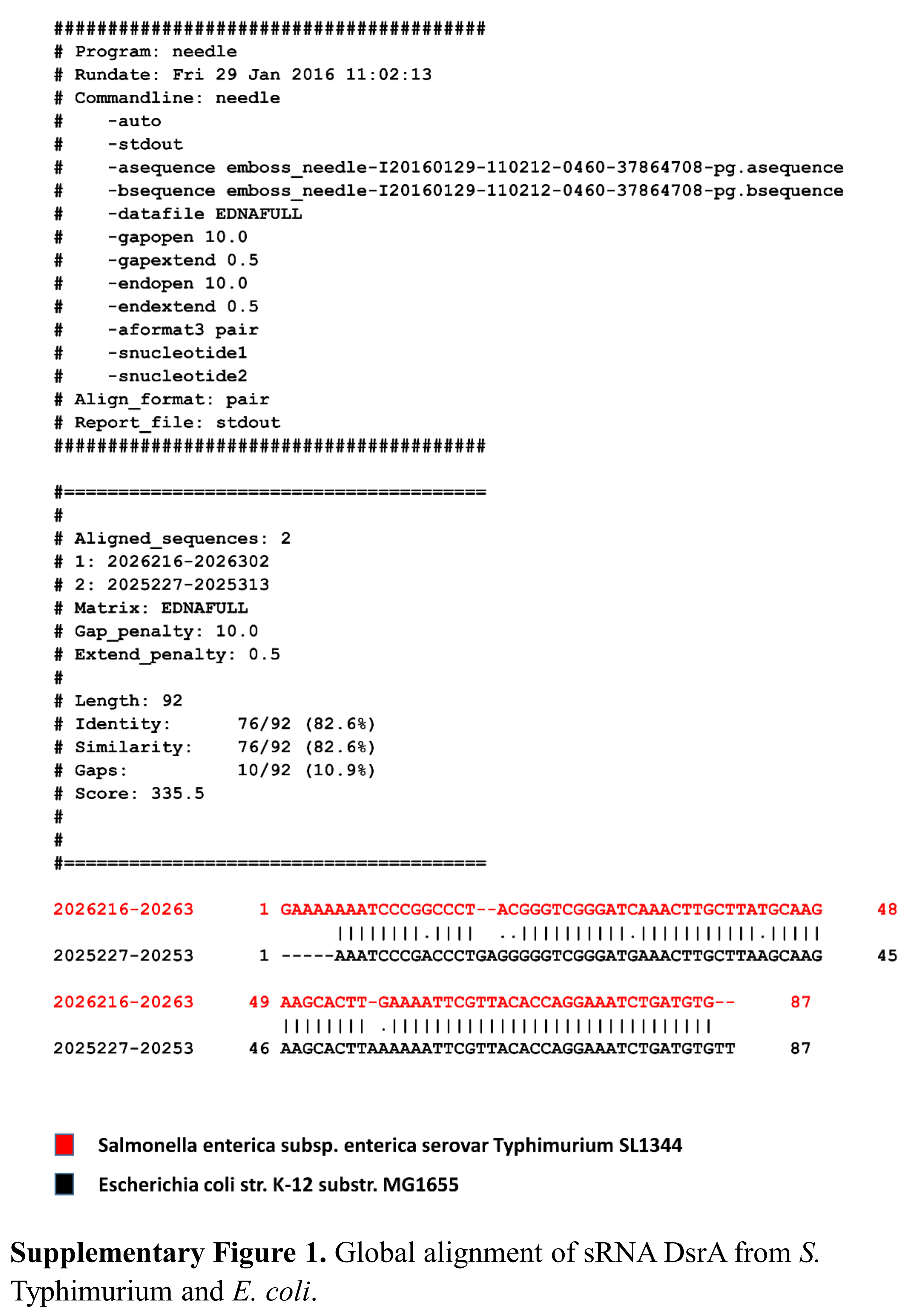

Supplement: Supplementary file 1 [file Image_1.TIF]
